# Evolution of malaria mortality and morbidity after the emergence of chloroquine resistance in Niakhar, Senegal

**DOI:** 10.1186/1475-2875-8-270

**Published:** 2009-11-27

**Authors:** Aline Munier, Aldiouma Diallo, Adama Marra, Michel Cot, Pascal Arduin, Ousmane Ndiaye, Balla Mbacké Mboup, Barnabé Gning, Jean-Philippe Chippaux

**Affiliations:** 1 IRD, UR010 Mother and Child Health in the Tropics, Université Paris Descartes, 4 av de l'Observatoire, 75270 Paris cedex 06, France; 2 IRD, UMR198 Emergent infectious and tropical diseases, Dakar, BP 1386, Senegal; 3 Fatick Medical Region, Fatick, Senegal; 4 Fatick Health District, Fatick, Senegal

## Abstract

**Background:**

Recently, it has been assumed that resistance of Plasmodium to chloroquine increased malaria mortality. The study aimed to assess the impact of chemoresistance on mortality attributable to malaria in a rural area of Senegal, since the emergence of resistance in 1992, whilst chloroquine was used as first-line treatment of malaria, until the change in national anti-malarial policy in 2003.

**Methods:**

The retrospective study took place in the demographic surveillance site (DSS) of Niakhar. Data about malaria morbidity were obtained from health records of three health care facilities, where diagnosis of malaria was based on clinical signs. Source of data concerning malaria mortality were verbal autopsies performed by trained fieldworkers and examined by physicians who identified the probable cause of death.

**Results:**

From 1992 to 2004, clinical malaria morbidity represented 39% of total morbidity in health centres. Mean malaria mortality was 2.4‰ and 10.4‰ among total population and children younger than five years, respectively, and was highest in the 1992-1995 period. It tended to decline from 1992 to 2003 (Trend test, total population p = 0.03, children 0-4 years p = 0.12 - children 1-4 years p = 0.04- children 5-9 years p = 0.01).

**Conclusion:**

Contrary to what has been observed until 1995, mortality attributable to malaria did not continue to increase dramatically in spite of the growing resistance to chloroquine and its use as first-line treatment until 2003. Malaria morbidity and mortality followed parallel trends and rather fluctuated accordingly to rainfall.

## Background

While childhood mortality is decreasing in Africa, malaria-specific mortality remains an alarming public health issue [1,2]. Moreover, resistance to chloroquine (CQ), which emerged in the 1980s, was considered responsible for a dramatic increase of malaria-specific mortality since the 1990s [3]. In the Senegalese Demographic Surveillance Site (DSS) of Niakhar, located in the region and department of Fatick, Trape *et al *[3,4] showed that malaria mortality among children of less than five years of age increased from 5.4‰ in 1988-1991 to 12.4‰ in 1992-1995, and attributed it to the emergence of CQ resistance in 1992.

**Figure 1:**
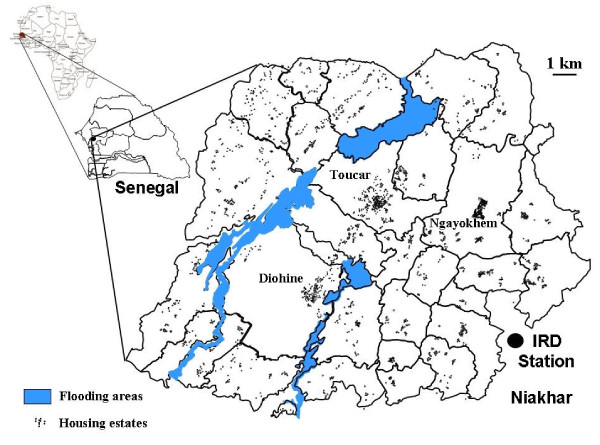
**Niakhar study area**.

In the DSS of Niakhar, *in vivo *tests conducted in 1993, 1994 and 1995 in Diohine village (Figure 1) showed 10-17% resistance rates of RII and RIII types at day 7 after CQ treatment, and 30-42% resistance rates at day 14 [5]. In other villages in the region of Fatick, 80 km from Niakhar DSS, Pradines *et al *[6] (1995) found 29% of all isolated strains resistant *in vitro *to CQ. In 1996, Sokhna *et al *[7] observed an *in vivo *resistance of 44% measured at day 14, in Diohine village. Additional studies conducted in a sentinel site located in the region of Kaolack, 60 km from Niakhar DSS, showed increasing *in vivo *resistance rates from 26% in 1998 to 42% in 2001 at day 14 [8].

The Senegalese national policy only replaced CQ as first-line treatment of malaria by the association amodiaquine/sulphadoxine-pyrimethamine (AQ/SP) in 2003. Meanwhile, the follow-up of the DSS of Niakhar population continued, aiming to describe and investigate the causes of mortality in the population, including children, in order to provide demographic and health data, and recommend preventive strategies.

This study attempts to evaluate retrospectively the influence of chemoresistance on the mortality attributable to malaria, mainly during the period 1992-2003 during which CQ resistance increased, whilst CQ remained the first-line treatment of malaria.

## Methods

### Study area and population

The Niakhar DSS, located in the region and the department of Fatick, 130 km south-east of Dakar in Senegal [9], is a demographic surveillance site since 1963, currently composed of 30 villages (Figure 1). It covers 230 square km and counted an average of 30,000 inhabitants between 1992 and 2004. Demographic (births, deaths, marriages, migrations) and various data (child vaccinations, economic incomes, child education, and daily rainfalls) were collected by permanent fieldworkers.

Three dispensaries situated in the villages of Diohine, Toucar and Ngayokhem, carried out basic health services to the population, including curative, preventive and prenatal care, birth deliveries, and vaccination. A nurse was in charge of the consultations and was assisted by a community health worker and a drug provider.

### Cause of death

It was assumed that the increase of mortality resulting from chloroquine resistance should be linked to proportional increase of malaria case-fatality rate. In the Niakhar DSS population, mortality is well addressed and causes of death are established through systematic and standardized verbal autopsy. Although data about deaths occurring among malarial patients consulting in health dispensaries were not available, malaria morbidity was estimated from the registers of dispensaries.

Other causes able to change the malaria transmission rate were also checked, including rainfall or interventions such as vector control in the studied area.

### Data collection

Death registration was performed by surveys consisting in domiciliary visits to all households of the DSS, conducted weekly from 1987 to 1996 and on a quarterly basis from 1997 onwards [10]. These surveys enabled fieldworkers to identify deaths occurring since their last visit. They informed the supervisor who sent an agent within the following three months in the specified households to realize a verbal autopsy. It consisted in a post-mortem investigation based on an interview conducted by the agent with the relatives of the deceased, usually one of the parents in case of a child's death. A standardized questionnaire containing identification of the person, history of symptoms and illness, and declared cause of death by the family, was completed by the fieldworker and subsequently reviewed by two physicians independently [11], who each identified the most probable cause of death. In case of discordant diagnosis, a group of three or four physicians discussed the cases to reach a consensus. Causes of death were coded using the International Classification of Diseases from the World Health Organization (WHO/ICD-9). The same methodology was used during the entire period of the study, regarding verbal autopsy questionnaire and attribution of death causes.

Morbidity data were drawn from health records of the three health care facilities. The records were completed by the nurse during the consultation, indicating the name of the patient, age, sex and village of residence, observed symptoms, clinical diagnosis and prescribed treatment. Registers from 1992 to 2004 were collected retrospectively.

Malaria diagnosis mainly relied on clinical signs as health centres lacked laboratory facilities or rapid diagnostic tools. Thus, terms of "malaria diagnosis" or "malaria morbidity" here refer to presumptive malaria not confirmed parasitologically. "Fever" is reported on the health record by the nurse, independently from the measure of temperature, whereas the "body temperature" is the temperature in Celsius degrees, measured by the nurse with a thermometer during the consultation.

Daily rainfalls were provided by a pluviometer located in the village of Niakhar, at the station of the IRD research institute and entered in Excel^® ^2003 (Microsoft Corporation, Redmond, WA, USA).

### Data processing and analysis

Morbidity data from health records were entered in dBase IV^® ^(dataBased Intelligence, Inc., Vestal, NY, USA). Annual number of consultations was reported to person-year (py) at risk in the study zone (expressed in number of consultations per 1,000 inhabitants) from 1992 to 2004. These demographic data were provided by the original Niakhar DSS database used for the prospective follow-up of the population.

Mortality data were entered in the database (dBase IV^® ^until 2003, and Access^® ^from 2003, Microsoft Corporation, Redmond, WA, USA) and analysed by the IRD team in Dakar.

Statistical analyses were performed using Stata 8.0^® ^software, StataCorp LP, Texas, USA. The comparison of quantitative variables was made by the Student t test. Proportions between groups (malaria diagnosis vs. non malaria) were compared using univariate logistic regression. Odds-ratio (OR) were given with their 95% confidence intervals (CI). Trends in malaria mortality between the three periods (1992-1995, 1996-1999, and 2000-2003) were analysed using the Cuzick trend test. Statistical significance was set to p = 0.05.

## Results

### Morbidity

A total of 110,093 consultations were reported between 1992 and 2004 in the three health care facilities. Malaria was clinically diagnosed in 43,232 patients (39.3%).

#### Characteristics of the patients

Overall male/female ratio was 0.83, females representing 54.7% of patients, but masculine gender was predominant before 15 years of age. Mean age was 17.5 years (median = 9 years, difference between genders: male = 6 vs. female = 13).

Children under 15 represented 56.8% of the consultations. Regarding consultations for malaria, 1-4 year-old children consulted in a greater number, followed by 0-1 year, 5-14s, 15-54s and, finally, above 54 years of age. Children aged one to 14 had an increased risk to be diagnosed malaria than another diagnosis by the nurse (1-4 years-old: OR = 1.34 (CI: 1.31-1.38), 5-14 years-old: OR = 2.21 (CI: 2.15-2.28)). The rainy season (from August to November) accounted for 65% of malaria cases, with 27% in October alone.

Positive predictive signs of a clinical diagnosis of malaria by the nurse were fever (OR = 5.23, IC [5.08-5.40], malaria vs. non-malaria diagnosis), convulsions (4.24 [3.57-5.04]), malnutrition (5.29 [4.99-5.60]), vomiting (5.62 [5.42-5.83]), headache (10.04 [9.70-10.39]) and chills (29.5 [25.6-34.0]). The mean body temperature was significantly higher among patients with a clinical diagnosis of malaria than non-malarial patients (38.0 vs. 37.2°C, p < 10^-5^).

#### Evolution of morbidity and rainfalls between 1992 and 2004

**Figure 2:**
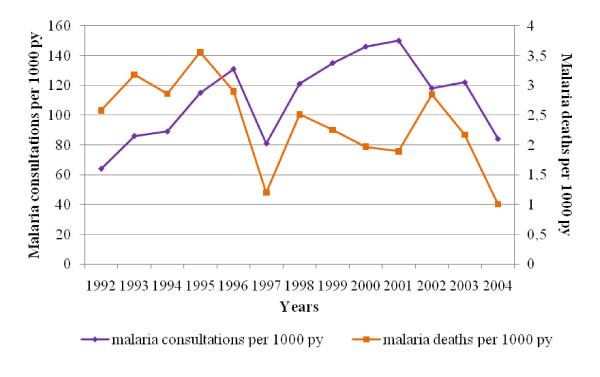
**Evolution of consultations for malaria in health care facilities and deaths attributable to malaria in Niakhar area (1992-2004)**. The purple line represents the clinical malaria morbidity in dispensaries per 1,000 person-year at risk (py). The orange line represents the malaria mortality per 1,000 py in the population.

Malaria and non-malaria morbidity followed similar trends over the time. Mean of all cause morbidity was 284‰ persons-years between 1992 and 2004, malaria and non-malaria counting respectively for 112‰ and 172‰. Malaria cases reached 150‰ in 2001, representing 49% of total morbidity that year, and fell to 84‰ in 2004. A drop in the total number of consultations occurred in 1997 (230‰), mainly attributed to a decrease in malaria-specific morbidity (81‰) (Figure 2).

**Figure 3:**
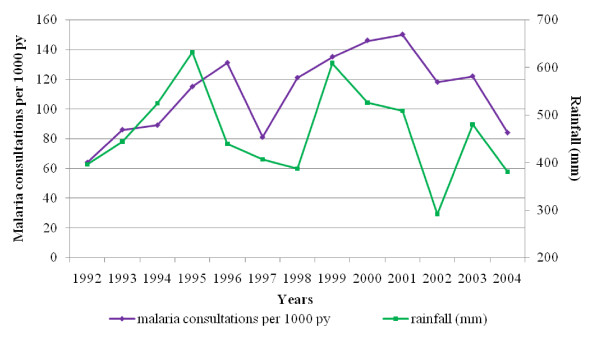
**Malaria consultations and annual rainfall in Niakhar area, 1992-2004**. The purple line represents the clinical malaria morbidity in dispensaries per 1,000 person-year at risk (py). The green line represents the annual rainfall (mm) in Niakhar village.

The average rainfall between 1992 and 2004 was 463.5 mm per year. From 1992, rainfall increased to reach a maximum in 1995 (631.7 mm) (Figure 3). In 1997, a drop of rainfall preceded the fall in malaria morbidity. Besides, between 1999 and 2001, consultations for malaria reached a maximum (mean 144‰), while rainfall was at a high level (mean 547.8 mm).

### Mortality

Overall and malaria-specific annual mortality were respectively 15.1‰ and 2.4‰. A peak in overall mortality occurred in 1998 (23.6‰), then it decreased until 2004 (9.6‰). Mortality attributable to malaria followed the same general tendency, but presented two peaks, in 1995 (3.6‰) and 2002 (2.9‰). Malaria mortality fell to 1‰ in 2004 (Figure 2).

Mean mortality attributable to malaria among children aged 0-4, 5-9 and 10-14 years was respectively 10.4‰, 2.2‰ and 0.3‰, between 1992 and 2004, and counted for 26.6%, 37.1% and 10.3% of overall mortality in those age groups (Table 1).

**Table 1:** Overall mortality and mortality attributable to malaria (deaths per thousand person-year at risk) in Niakhar study area population - all children, 0-4, 5-9 and 10-14 years - from 1992 to 2004.

	Total < 15 years		Children		Children		Children	
			0-4 years		5-9 years		10-14 years	
**Years**	**Overall**	**Malaria**	**Overall**	**Malaria**	**Overall**	**Malaria**	**Overall**	**Malaria**
	**mortality**	**mortality**	**mortality**	**mortality**	**mortality**	**mortality**	**mortality**	**Mortality**
	**(‰)**	**(‰)**	**(‰)**	**(‰)**	**(‰)**	**(‰)**	**(‰)**	**(‰)**

1992	21.5	5.08	37.4	10.1	5.5	2.8	2.1	0.30
1993	22.4	6.32	44.4	13.2	5.4	3.2	3.5	0
1994	17.3	5.34	43.6	10.3	6.6	3.7	2.2	0.28
1995	19.2	7.46	33.9	16.3	6.0	3.4	0.3	0
1996	20.1	6.13	72.7	14.0	6.8	1.9	4	0.53
1997	15.4	2.51	65.9	4.7	5.2	2.2	2.7	0
1998	33.0	5.27	46.3	11.6	11.5	2.6	6.8	0.24
1999	29.2	4.87	35.3	12.1	10.5	1.3	5.3	0
2000	20.7	4.3	32.7	9.8	7.9	2.0	3.8	0.23
2001	15.2	3.86	30.2	9.0	4.1	1.1	2.1	0.46
2002	14.1	5.61	18.7	11.9	3.7	2.4	1.6	1.15
2003	13.1	4.12	30.2	8.5	2.2	1.7	2.3	0.91
2004	8.5	1.84	18.7	4.3	1.9	0.4	1.6	0

*mean 1992-2004*	*19.2*	*4.8*	*39.2*	*10.4*	*5.9*	*2.2*	*2.9*	*0.30*

**Figure 4:**
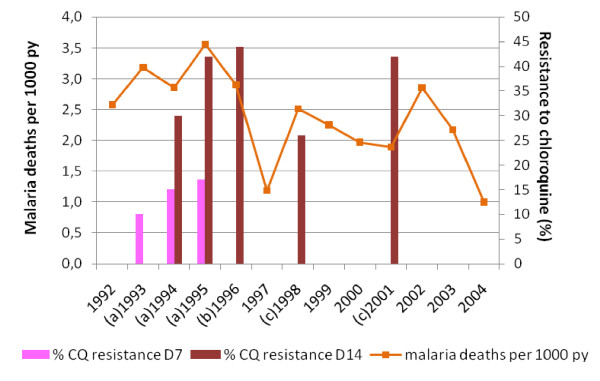
**Evolution of malaria mortality and resistance to chloroquine between 1992 and 2004 in Niakhar area**. The orange line represents the malaria mortality per 1,000 person-year (py) in the population. Red bars represent the rate of chloroquine resistance (%) measured at day 14 after treatment by chloroquine. Pink bars represent the rate of chloroquine resistance (%) measured at day 7 after treatment by chloroquine. (a) 1993, 1994, 1995: *in vivo *chemosensitivity studies, Diohine village, Niakhar DSS [5]. (b) 1996: *in vivo *chemosensitivity study, Diohine village, Niakhar DSS [7]. (c) 1998 and 2001: *in vivo *chemosensitivity studies, Kaolack sentinel site, 50 km from Fatick [8].

Considering the three consecutive periods 1992-1995, 1996-1999 and 2000-2003, there was a significant downward trend in malaria mortality among all the population (children and adults) (Cuzick trend test, p = 0.031). Among children less than 15 years, malaria mortality presented a declining trend, although not significant (p = 0.062). It was respectively 12.5‰, 10.6‰ and 9.8‰ for 0-4 years (p = 0.117). Concerning more particularly children aged 1 to 4 years, it was respectively 13.3‰, 11.7‰ and 9.0‰ (p = 0.039). It was 3.3‰, 2.0‰ and 1.8‰ for 5-9 years (p = 0.011) and 0.2‰, 0.2‰ and 0.7‰ for 10-14 years (p = 0.09). In 2004, malaria mortality decreased to 4.3‰, 0.4‰ and 0.0‰ in each age group respectively. Although CQ resistance was continuously growing during the period of study, malaria mortality tended to decline in the area (Figure 4).

## Discussion

Chloroquine resistance is considered as the first cause of increasing risks of malaria morbidity and mortality in the world [3,4,12,13]. Two causes could explain why chemoresistance could induce an increase of consultations for malaria in health centres. The very first cause is likely to be the failure of self-medication that is a common behaviour in the Niakhar population in order to treat fever before going either to traditional healer or modern dispensary [14,15]. However, anti-malarial drugs represented only 18.2% of total self-medication versus 64.7% for antipyretics [15] and chloroquine was seldom available on markets even though it was a cheap and affordable drug. The second cause is likely to be the failure of chloroquine treatment administered by nurses of health centres. The emergence of CQ resistance results in the return of non-cured patients for further care within the few days or weeks following the CQ treatment. One could hypothesize that returns of patients treated with chloroquine should increase proportionally to the CQ resistance. Nevertheless, an ancillary study conducted in the same dispensaries, did not show increasing rates of returns after CQ treatment during this period [16].

Morbidity data used in this study were based on clinical malaria which is known to overestimate the number of malaria cases [17-20]. The main clinical criteria for diagnosing malaria did not change over the time in two of the health centres -representing 84% of total morbidity data- which allowed to study the main trends. These data indicate a consistency in the practices of health staff. Predictive signs of clinical diagnosis of malaria, such as age of patient (1-14 years), presence of fever, headache and vomiting, and treatments prescribed (varying with the diagnosed pathology), showed that nurses followed a continuous rationale in order to distinguish malaria from other diseases.

The overall morbidity fluctuated over the period 1992-2004, showing two peaks in 1996 and 1999, respectively attributed to a cholera outbreak and both meningitis and shigellosis epidemics [11]. In 1997, the drop in consultations for malaria could be linked to the lower rainfall and lower malaria transmission in the area.

Other causes than an increase in morbidity in the population of the area could actually explain a higher attendance at the dispensary. For example, the persistence of the same nurse since 1996 in one of the public health facilities may have played an essential role in health-seeking behaviour of patients, probably contributing to increase the activity of this centre. Valin *et al *[21] demonstrated in the same zone that a follow-up of populations and a free access to health care with an early treatment of malaria cases were key elements in the control of malaria, even in a zone of high resistance to CQ.

While morbidity concerned only patients consulting in the dispensaries of the DSS, mortality was measured in the entire population and should be considered as exhaustive due to the collecting methods. The causes of deceases, including malaria, were determined by verbal autopsies (VA) that give the most probable cause of death, estimated by physicians according to a standardized questionnaire and procedure, indicating the context of death and the history of the disease, and filled in by a trained fieldworker during his interview with the relatives of the deceased. It was shown that validity of VA strongly depended on the pathology responsible for the death [22]. According to a study by Snow *et al *[23] in Kenya, VA specificity was higher than 80% for the main causes of death; sensitivity varied from good (>75%) for diseases such as measles, neonatal tetanus, malnutrition and accidents, to moderate (<50%) for other pathologies, such as malaria, anaemia and respiratory infections. Specificity and sensitivity of VA compared to a diagnosis made at hospital level, concerning malaria, were respectively 89% and 46%. Chandramohan *et al *[24] showed similar results with a lower sensitivity of malaria-specific VA (33% and 19% for VA interpreted respectively by a physician or a diagnosis algorithm).

Nevertheless, in the absence of an effective national death register at the population level, and for rural regions where most deaths occur at home, the VA method proved to be a satisfactory tool, and the only one that can currently overcome the lack of reliable data on mortality causes. In the Niakhar DSS, VA methodology was constant throughout the period of study, using standardized questionnaires and causes of deaths being identified by at least two physicians, which allows considering that it is a valid tool for trend computation.

However, periodicity of the collection of demographic events -including deaths- changed from weekly to quarterly in 1997, due to the end of vaccine trials that were conducted in the study area during the previous period (1987-1996). This might have led to underreporting of deaths explaining the low figures in malaria mortality that same year. Nevertheless, morbidity figures, from a different source of data -i.e. dispensaries- showed the same drop in 1997, and rainfall level was also low and could have induced a lower malaria transmission.

Tests of trends in malaria mortality were repeated, excluding the year 1997, and showed similar results (downward trend, children 0-4 years p = 0.088; children 1-4 years p = 0.025, children 5-9 years p = 0.014).

Chemoresistance may induce a rise in malaria-specific mortality, resulting from therapeutic failures -as suggested by Trape *et al *[3,4] until 1995. Following this hypothesis, after 1995 malaria mortality would have been expected to remain at a high level, or even to increase accordingly to the chemoresistance rate, as long as CQ would have been still used for malaria treatment.

Mortality attributable to malaria presented two peaks in 1995 and 2002 and revealed a maximum of deaths during the first period of the study (1992-1995). Studies conducted by Trape *et al *[4] showed that malaria mortality among children aged 0-9 years doubled between the 1988-91 and 1992-95 periods. Using the same methodology (verbal autopsies) and sources of data (Niakhar database), it is shown in this study that the increase of mortality reversed after 1995, although CQ resistance continued to grow. Between 1992 and 2003, it has been previously observed that CQ remained the first-line treatment against malaria in the three health centres of Niakhar. CQ was widely prescribed in parallel with malaria morbidity in spite of the CQ resistance. Prescriptions declined slightly at the beginning of 2000s in favour of quinine or SP, and CQ was officially replaced by the AQ/SP combination in 2003 [25]. The maintenance of the presumed efficacy of chloroquine to treat malaria is difficult to explain. It may be hypothesized that chloroquine was able to lower the parasitaemia sufficiently in most of the patients to allow controlling the disease - at least in its serious forms. Moreover, quinine was used improperly, i.e. not only to patients suffering severe malaria; it was prescribed either alone or in combination with chloroquine, and could have hidden the lack of efficacy of CQ. Nevertheless, quinine was not used increasingly from 1992 to 2001, while CQ resistance rose.

Etard *et al *[11] also showed that although some causes of deaths, in particular diarrhoea and acute respiratory infections, had risen in children younger than five years between 1995-97 and 1998-2000, malaria-specific deaths did not participate in the overall increase in mortality.

The high malaria mortality observed in 1992-1995 can also be partly attributed to the inappropriate treatment of cerebral malaria carried out in dispensaries at this time. Intravenous quinine was diluted with saline, which could induce severe hypoglycaemia leading to death. Since 1995, isotonic glucose replaced saline in the treatment of severe malaria.

Morbidity and mortality come from two different sources of data -dispensaries vs. entire DSS population, but are consistent with each other. Without any information on case-fatality rates, morbidity data were used, hypothesizing that, if the case-fatality rate increased with chemoresistance, mortality rates would be susceptible to increase faster than morbidity rates. Conversely, parallel trends were observed for both indicators from 1992 to 1998, and a discrepancy occurred from 1999 to 2001, with an increase in morbidity concomitant with a decrease in mortality (Figure 2).

Thus, malaria morbidity and mortality did not follow the increasing CQ resistance (Figure 4). Both appeared to be closely related to rainfall (Figure 3). Robert *et al *[26] showed that malaria transmission was unstable in this mesoendemic area, with a low annual entomological rate and mainly dependent on rainfall and water level variations in local temporary ponds. Ndiaye *et al *[27] demonstrated a significant relation between climate variability in August and malaria mortality from August to December in Niakhar area between 1984 and 1996. It may be pointed out that, together with the highest level of mortality attributable to malaria, rainfall increased gradually from 1992 to 1995.

Lastly, in 2004, mortality attributable to malaria dropped, following the fall of morbidity. Although it can be explained by the new anti-malarial policy that recommended amodiaquine/sulphadoxine-pyrimethamine as first-line treatment, rainfall was low (380.9 mm) and could also have contributed to the decline in mortality.

During the period of study, other factors susceptible to have an impact on malaria morbidity and mortality were not present or trivial, except an IPTi (Intermittent Preventive Treatment in infants) trial conducted by Cisse in 2002, which affected 535 children aged 2-59 months (less than 10% of the whole DSS population of children aged<5 years) treated with one dose of AQ/SP [28,29]. Furthermore, mortality rates had started to decline since 1996, i.e. six years before the beginning of this trial. Effective control measures were not widely developed, with uncommon insecticide-treated nets (ITN) usage and no indoor residual spraying implementation. The Demographic and Heath Survey conducted in Senegal in 2005 (DHS-IV [30]) showed that only 5% of children under five years of age slept under an ITN the night preceding the survey, in the Fatick region.

Finally, no major change in socio-economic status of residents, or in patients' health-seeking behaviour occurred, that could have impacted malaria morbidity and mortality.

## Conclusion

Malaria appeared to be the main cause of deaths among children below 15 years of age (25% of total deaths). The number of deaths attributable to malaria increased from 1992 to 1995, as stated by Trape *et al *[4] but stabilized and declined thereafter, in spite of the use of chloroquine as first-line treatment until 2003, showing the necessity of a longer surveillance before assessing the development of morbidity and mortality of malaria after the emergence of antimalarial resistance.

Morbidity and mortality attributable to malaria presented similar trends over the time and were concordant with rainfall rather than chemoresistance patterns. Thus, while the impact of chloroquine resistance on malaria mortality is probable, it is certainly not the only cause nor even the predominant cause, at least in this mesoendemic malarial area.

New national policy regarding malaria treatment from 2004 and reliable diagnosis of malaria using routine rapid diagnosis tests in health centres from 2007 should be helpful for the malaria surveillance and the understanding of the causes of morbidity and mortality fluctuations. Although chemoresistance must remain a concern in order to improve the management of malaria, other factors, such as climate and those affecting risks of malaria transmission, should also be considered.

## Competing interests

The authors declare that they have no competing interests.

## Authors' contributions

AM participated in the study design, data collection, analysis and interpretation, and drafted the manuscript. AD contributed to the study design, data collection and interpretation, and revision of the manuscript. AMa contributed to the study design and supervision, and update and analysis of Niakhar DSS database. MC participated in data interpretation, writing, and critical review of the manuscript. PA contributed to the study design, supervision of data collection, and revision of the manuscript. ON participated in the supervision of data collection, and designed the data entry screen for health records collection. He participated to the revision of the manuscript. BMM and BG participated in data interpretation and revision of the manuscript. JPC was the conceptor of the study; he contributed to data interpretation, preparation and review of the manuscript. He is the guarantor of the paper. All authors read and approved the final manuscript.
